# Environmental stress and epigenetic transgenerational inheritance

**DOI:** 10.1186/s12916-014-0153-y

**Published:** 2014-09-05

**Authors:** Michael K Skinner

**Affiliations:** Center for Reproductive Biology, School of Biological Sciences, Washington State University, Pullman, WA 99164-4236 USA

**Keywords:** Epigenetic, Non-genetic inheritance, Stress, Transgenerational

## Abstract

Previous studies have shown a wide variety of environmental toxicants and abnormal nutrition can promote the epigenetic transgenerational inheritance of disease. More recently a number of studies have indicated environmental stress can also promote epigenetic alterations that are transmitted to subsequent generations to induce pathologies. A recent study by Yao and colleagues demonstrated gestational exposure to restraint stress and forced swimming promoted preterm birth risk and adverse newborn outcomes generationally. This ancestral stress promoted the epigenetic transgenerational inheritance of abnormalities in the great-grand offspring of the exposed gestating female. Several studies now support the role of environmental stress in promoting the epigenetic transgenerational inheritance of disease. Observations suggest ancestral environmental stress may be a component of disease etiology in the current population.

Please see related article: http://www.biomedcentral.com/content/pdf/s12916-014-0121-6.pdf.

## Background

The ability of environmental factors, such as stress [1], to promote the epigenetic transgenerational inheritance of disease and phenotypic variation has now been established in a number of organisms ranging from plants to humans, with a variety of environmental exposures [2]. One of the first studies found that environmental toxicants such as fungicides and pesticides promoted epigenetic transgenerational inheritance of reproductive disease [3]. Subsequently a large number of different types of toxicants (plastics, hydrocarbons, dioxin, biocides, dichlorodiphenyltrichloroethane (DDT)) have been shown to promote the transgenerational inheritance of disease [4] from obesity to cancer [5] (Table 1). Other critical environmental factors found to promote transgenerational disease are nutritional abnormalities such as caloric restriction or high fat diets [6]. In species such as insects and plants both drought and temperature have also been shown to be critical environmental factors [7,8] (Table 1). Therefore, a large number of environmental factors have been shown to promote the epigenetic transgenerational inheritance of disease or phenotypic variation in a variety of different species, including humans [9]. This environmentally induced form of non-genetic inheritance will have a significant impact on disease etiology [2,10] and areas of biology such as evolution [11].

**Table 1:** **Examples of transgenerational inheritance studies**

**Exposure**	**Pathology**	**Reference**
**Toxicants**		
Vinclozolin	Testis, prostate, kidney disease, tumors, immune	Anway *et al.*, 2005 [3]; 2006 [12]
	Gender-specific changes in anxiety-like behavior	Skinner *et al.*, 2008 [13]
	Immune and reproductive	Nilsson *et al.*, 2008 [14]
Methoxychlor	Testis, kidney, ovary, obesity	Anway *et al.*, 2005 [3], Manikkam *et al.* 2014 [15]
Permethrin/DEET	Prostate, kidney disease	Manikkam *et al.* 2012 [16]
Dioxin	Prostate, kidney, fertility, pregnancy	Manikkam *et al.* 2012 [17] Bruner-Tran *et al.* 2011 [18]
BPA/phthalates	Prostate, kidney, obesity	Manikkam *et al.* 2013 [19]
Hydrocarbon mixture (jet fuel)	Prostate, kidney, obesity, immune and reproduction	Tracey *et al.* 2013 [20]
Vinclozolin, permethrin/DEET, plastics, dioxin, jet fuel	Polycystic ovaries, reduced primordial follicle pool	Nilsson *et al.* 2012 [21]
DDT	Obesity, kidney, testis	Skinner *et al.* 2013 [5]
Phthalate	Testis and spermatogonial stem cell	Doyle *et al.* 2013 [22]
Tributyltin	Obesity and adipose cell	Chamorro-Garcia *et al.* 2013 [23]
BPA	Social behavior, implantation, litter size, sperm	Wolstenholme *et al.* 2012 [24]; Salian *et al.* 2009 [25]
**Others**		
Caloric restriction	Cardiovascular mortality	Bygren *et al.* 2014 [26]
High fat diet	Growth and insulin sensitivity	Dunn and Bale 2011 [6]
Folate	Congenital malformations	Padmanabhan *et al.* 2013 [27]
Drought	DNA methylation changes	Zheng *et al.* 2013 [7]
Heat/salt	Flowering and salt tolerance	Suter and Widmer 2013 [28]
Prediabetes	Glucose tolerance and insulin sensitivity	Wei *et al.* 2014 [29]
Smoking	Abnormal pulmonary function	Rehan *et al.* 2013 [30]
Alcohol	Endocrine and neuronal function	Govorko *et al.* 2012 [31]
Heat stress	Increased Hsp70 production and tolerance to heat stress	Norouzitallab *et al.* 2014 [8]

BPA, Bisphenol A; DEET, N,N-diethyl-m-toluamide.

**Figure 1:**
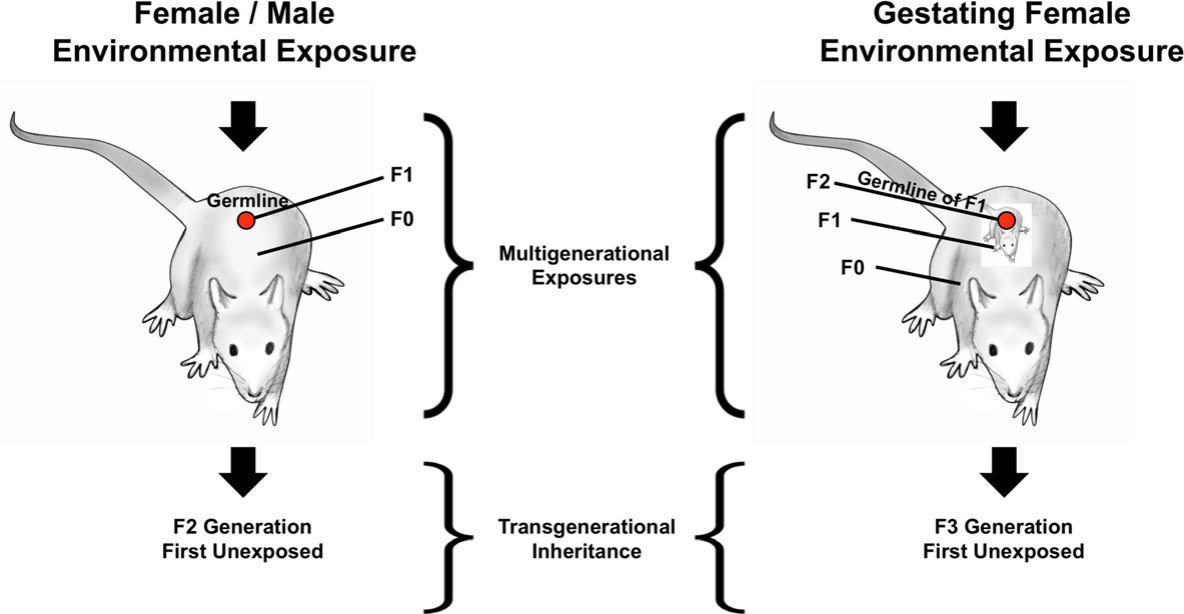
**Schematic of multigenerational exposure and transgenerational inheritance.**

Epigenetic transgenerational inheritance is defined as ‘the germline (egg or sperm) transmission of epigenetic information between generations in the absence of any environmental exposure’ [10]. Direct environmental exposure does not involve a generational phenotype, only direct toxicity or physiological effects of the individual exposed [2]. As previously described [2,32], the exposure of an individual any time during development (F0 generation) results in the exposure of that individual and the germline (sperm or egg) that will generate the next generation (F1 generation) (Figure 1). The exposure of a gestating female exposed the F0 generation female, F1 generation fetus and germline that will generate the F2 generation (Figure 1). The ability of an exposure to act on multiple generations is termed a multigenerational exposure [32]. Where direct exposure is involved, no transgenerational effects are observed. Unfortunately, many studies have misused the term transgenerational to refer to multigenerational exposure effects. By contrast, if studies are extending to generations with no direct environmental exposure then observed effects can be considered transgenerational because the germline is the only cell type able to transmit epigenetic information generationally (Figure 1).

Epigenetics is defined as ‘molecular factors/processes around DNA that regulates genome activity independent of DNA, and that are mitotically stable’ [10]. The types of molecular processes involved are DNA methylation, histone modifications, chromatin structure, and non-coding RNA (ncRNA). The best characterized epigenetic factor to be involved in germline transmission of epigenetic information is DNA methylation. An example is imprinted genes that mediate paternal or maternal allelic transmission of specific DNA methylation patterns [33]. A number of studies have shown that environmentally induced epigenetic transgenerational inheritance involves altered germline DNA methylation [4,34]. More recently ncRNA has been suggested as an additional mechanism in germline transmission of epigenetic information [35]. Histone modifications have also been suggested in a variety of organisms [36]. Although DNA methylation has a critical role in fetal germline development and early embryonic development [37], all the epigenetic processes will likely be involved and have unique functions in regulating development [10]. Further studies regarding the role of all epigenetic processes in environmentally induced epigenetic transgenerational inheritance are required.

## Environmental stress and transgenerational phenotypes

A number of studies have shown multigenerational effects of stress [38]. One of the best initial examples was the work of Suderman and colleagues [39] showing the generational effects of maternal care on early postnatal life. Optimal early postnatal maternal care promoted epigenetic programming of the brain that created an adult female with good maternal care characteristics, which then passed on to subsequent generations. By contrast, bad early postnatal maternal care (environmental stress) promoted bad maternal characteristics later in life and altered epigenetic programming of the brain to propagate bad maternal care generationally [39]. This is a good example of an environmental exposure at each generation promoting epigenetic programming that leads to a specific phenotype in the individual, that is, a multigenerational exposure [32]. Other examples of multigenerational exposures influenced by stress have also been described [40–42]. Environmentally altered epigenetics is the critical molecular mechanism for these multigenerational exposures [32]. Somatic cell epigenetic effects will be the most predominant environmental impacts on an individual’s phenotype and disease. If these effects do not involve the germline, they will not be transmitted to subsequent generations.

One of the initial studies to demonstrate environmental stress promoting the epigenetic transgenerational inheritance of disease was a three-generation study involving maternal separation and maternal restraint stress [43]. Social abilities and brain function showed transgenerational alteration in the F2 and F3 generations. A recent study investigated the ability of a paternal olfactory stress experience to promote the transgenerational inheritance of an olfactory stress response in F2 generation progeny [44]. Correlations with DNA methylation patterns in the olfactory receptor system were documented in the transgenerational offspring. Although a limited number of transgenerational stress-induced pathologies have been observed (Table 2), there have been reviews on the topic [38,45].

**Table 2:** **Stress-induced transgenerational inheritance of pathologies**

**Stress exposure**	**Pathology**	**Reference**
Maternal separation and stress	Social anxiety and recognition and stress resilience	Franklin *et al.* 2011 [43]
Traumatic paternal stress (odorant)	Behavioral and neural metabolic responses	Dias *et al.* 2014 [44]
Gestational restraint and forced swimming	Preterm birth and prenatal growth and behavior	Yao *et al.* 2014 [1]

In addition to the ability of ancestral stress to induce the epigenetic transgenerational inheritance of disease, a previous study demonstrated altered stress responses in transgenerational individuals [46]. Toxicant (vinclozolin) lineage transgenerational (F3 generation) rats were found to have altered stress responses (adolescence restraint stress) later in life. These stress responses were sex specific and gene expression networks in brain regions were found to correlate with these transgenerational stress responses [47]. Therefore, stress can induce the transgenerational inheritance of disease, and ancestral exposures to a variety of factors can alter stress response transgenerationally.

## Ancestral stress exposure promotes preterm birth and newborn abnormalities

Yao and colleagues [1] designed a study to investigate the ability of environmental stress to promote the epigenetic transgenerational inheritance of disease. The experimental design exposed a gestating female to restraint stress and forced swimming in the later stages of fetal development. The offspring (F1 generation) were bred to generate F2 and F3 generations. A non-stress control lineage, stress lineage (only F0 generation female stress) and chronic stress lineage (all generations stressed) were examined for preterm birth and newborn abnormalities. The F3 generation stress lineage animals had decreased pup weights and altered developmental behaviors. The gestational length progressively declined with each generation leading to a higher preterm birth risk. The F2 generation brain and uterus expression of ncRNA for selected miRNA were altered. Therefore, the study demonstrated that gestational stress promoted the epigenetic transgenerational inheritance of preterm birth risk and decreased brain development of early postnatal offspring.

This is the first study to suggest ancestral stress can influence transgenerational preterm birth risk. Preterm birth in humans is linked to a number of postnatal abnormalities [48]. There has been a dramatic increase in preterm birth rates in recent years. Although there have been a number of proposed factors for this rise in preterm births, the current study of Yao and colleagues [1] suggests ancestral gestational stress may be a component in the pathology. Although further research is needed, the concept that ancestral gestational stress may have a role in promoting transgenerational preterm birth risk is a novel component of the disease etiology to consider. Similar considerations can be proposed for early postnatal neurodevelopmental abnormalities.

## Conclusions

The study of Yao and colleagues [1] supports a role of ancestral stress in the epigenetic transgenerational inheritance of disease. Although direct stress exposure of adults can influence pathologies in the individual and offspring, the multigenerational versus transgenerational inheritance characteristics of the pathology need to be considered. A direct exposure generally affects somatic tissues that will be critical for the individual’s disease, but a transgenerational effect requires a transmission of epigenetic information by the germline. Often, as shown in the current study [1], the transgenerational disease and pathology is distinct and/or has greater frequency than the direct exposure pathology [5]. The ability of stress to promote the epigenetic transgenerational inheritance of disease has now been shown in several different laboratories and animal model systems (Table 2).

A variety of environmental factors promote the epigenetic transgenerational inheritance of disease (Table 1). The observation that environmental stress can also promote transgenerational pathologies suggests ancestral stress conditions may be a significant factor in our own disease and what we pass down to our grandchildren. Several studies have considered the multigenerational impacts of stress on future generations, including World War 2 holocaust survivors’ offspring [49] and traumatic stress generational effects in several African countries [50,51]. The concept that ancestral stress, particularly during gestation, may influence disease etiology for generations to come is an important aspect to consider in regards to our environment and society. This is a novel concept that will need to be seriously considered in our future health management and therapy.
